# Spinal cord tethering and syringomyelia after trauma: impact of age and surgical outcome

**DOI:** 10.1038/s41598-023-38565-0

**Published:** 2023-07-15

**Authors:** Denis Bratelj, Susanne Stalder, Crescenzo Capone, Phillip Jaszczuk, Cristian Dragalina, Tobias Pötzel, Catherine Gebhard, Michael Fiechter

**Affiliations:** 1 Spine and Orthopedic Surgery, Swiss Paraplegic Center, Nottwil, Switzerland; 2 Department of Nuclear Medicine, University Hospital Zurich, Zurich, Switzerland

**Keywords:** Spinal cord diseases, Neurosurgery

## Abstract

Posttraumatic spinal cord tethering and syringomyelia frequently lead to progressive neurological loss. Although several studies demonstrated favourable outcome following spinal cord detethering with/without shunting, additional research is required as no clear consensus exists over the ideal treatment strategy and knowledge about prognostic demographic determinants is currently limited. In this investigation, we retrospectively investigated 67 patients (56 men, 11 women) who were surgically treated and followed for symptomatic spinal cord tethering and syringomyelia from 2012 to 2022 at our center. Age (B-coefficient 0.396) and severity of trauma to the spinal cord (B-coefficient − 0.462) have been identified as independent predictors for the rate of development of symptomatic spinal cord tethering and syringomyelia (p < 0.001). Following untethering surgery including expansion duraplasty with/without shunting, 65.9% of patients demonstrated an improvement of neurological loss (p < 0.001) whereas 50.0% of patients displayed amelioration of spasticity and/or neuropathic pain (p < 0.001). Conclusively, active screening for symptomatic spinal cord tethering and syringomyelia, particularly in younger patients with severe spinal trauma, is crucial as surgical untethering with/without shunting is able to achieve favourable clinical outcomes. This knowledge may enable clinicians to tailor treatment strategies in spinal cord injury patients suffering from progressive neurological loss towards a more optimal and personalized patient care.

## Introduction

The development of spinal cord tethering and syringomyelia after spinal cord injury (SCI) is a well-described phenomenon^1,2^. Posttraumatic ischemia of the spinal cord in combination with autolytic processes of intra-/extramedullary hematoma and subarachnoid scarring hampers cerebrospinal fluid (CSF) flow and may finally lead to the development of spinal cord tethering and formation of an intramedullary cyst also known as syringomyelia which is currently regarded as end point of a process^3,4^. In particular, progressive neurological loss due to a tethered cord with or without syringomyelia usually triggers surgical detethering with expansion duraplasty, and when indicated syrinx shunting^5–7^. In a consensus panel in 2010, a strong recommendation was proposed for surgical treatment in case of deteriorating motor function while there was only a weak recommendation for surgery in patient cases with progressive sensory loss or neuropathic pain^8^. Although large investigations exist with several hundred patient cases using consistent surgical approaches and displaying more than 5 years of postoperative follow up^5,9^, there is still no consensus on what the optimal standard treatment should be in this disabling pathology. Even if spinal cord tethering is diagnosed early in its course by modern diagnostic modalities such as high field strength magnetic resonance imaging (MRI), a surgical strategy is not necessarily claimed to be superior in comparison to a conservative therapy^10^. However, a high variability of surgical approaches in patients with symptomatic spinal cord tethering and syringomyelia presently renders it highly demanding to validate efficacy of a surgical strategy^11^. Further, in epidemiological studies with SCI cohorts, level and severity of injury significantly correlates with age and male predominance^12,13^. Interestingly, only scarce information is available about demographic discriminators in SCI patients suffering from symptomatic spinal cord tethering and syringomyelia. As this is a rather rare disease with an incidence of approx. < 1 to 7% concerning symptomatic patients^1^, it is of importance to identify independent risk factors which facilitates patient screening in clinical routine in order to early detect clinically relevant cases subject for treatment.

Given the controversies regarding the optimal treatment strategy in patients suffering from symptomatic spinal cord tethering and syringomyelia after trauma and the observed demographic disparities in SCI patients, we aimed to investigate demographic variability and treatment efficacy in a cohort of middle-aged patients who underwent a consistent surgical approach by spinal cord detethering and/or syrinx shunting in order to overcome inconsistencies and adding further evidence to the existing literature proclaiming surgical treatment strategies in this disabling disease.

## Materials and methods

### Patient population

All patients with a traumatic SCI who were either operated for posttraumatic spinal cord untethering and/or syringomyelia or were in regular follow up after such an intervention between 2012 and 2022 at the Swiss Paraplegic Center were included into this retrospective observational monocenter study. The included patients all underwent spinal cord detethering with expansion duraplasty in combination with or without cyst/syrinx shunting, comparable to the technique described by Falci et al.^4,5^. Indication for untethering surgery was given if the clinical symptomatology was functionally relevant for the patient and all other potential triggers mimicking a similar symptomatology had been excluded. After laminectomy and midline opening of the dura over the affected segments, the dorsal and lateral aspect of the spinal cord is deliberated from the scarring arachnoid web by lysis of the adhesions. In order to omit immediate retethering due to postsurgical inflammatory processes an enlargement of the intradural space by an expansion duraplasty is usually performed. The duraplasty is then attached to the surrounding tissue or stabilization material to ensure persistent enlargement of the intradural space. If during the untethering process the collapse of a present syrinx was less than 50% (as verified by intraoperative ultrasound), then a cyst/syrinx shunting was performed. In cases where the lower thoracolumbar region was spared of spinal tethering, a subarachnoid-subarachnoid shunt was regarded as sufficient while in cases where the lumbar region was affected by scarring/cysts a syringo-peritoneal shunt was implanted. Patients with insufficient clinical and/or imaging data or written denial of informed consent were excluded from the investigation (n = 15). Finally, 67 patients (56 men, 11 women) were further analyzed in this study. Regarding initial trauma leading to SCI, 40 patients (59.7%) had surgery at the level of injury (stabilization with decompression) with most injuries located at the upper to mid thoracic level (thoracic vertebra 4 to 5). The mean time interval from initial trauma to untethering surgery was 16.8 ± 13.4 years. The mean follow-up after surgical intervention was 8.1 ± 7.1 years (range 0.1 up to 30 years).

The study and its protocols have been approved by the local ethics committee (Ethikkommission Nordwest- und Zentralschweiz (EKNZ), KEK-2021-00890) which also waived the need for written informed consent. Further, all methods were performed in accordance with the relevant guidelines and regulations.

### Data collection

Patient relevant data was retrieved from the electronic clinical information system and the imaging database. Clinical symptoms which triggered surgical spinal cord detethering and/or shunting were qualitatively graded as present or absent and categorized by sensory (impairment of sensation to touch, vibration, and/or temperature feeling) and/or motor symptoms (functionally relevant decrease of motor strength in relevant key muscles), spasticity (presence of hypertonia in legs, arms, and/or trunk) and/or neuropathic pain (any type of non-nociceptive pain), or a combination of those symptoms. After surgery, clinical symptomatology was assessed as improved, stationary, or worsened according to patient records using the same clinical key symptoms as described above. Patients were classified by the use of the American Spinal Injury Association Impairment Scale (AIS) before and after surgical intervention. Finally, imaging data (MRI) was investigated by the extent of spinal cord tethering/syringomyelia (in vertebral segments) and qualitatively graded by the radiologist as improved, stable, or worsened after spinal cord intervention (in comparison to images before surgery).

### Statistical analysis

Quantitative variables are displayed as mean ± standard deviation (SD) and categorical variables as frequencies or percentages. SPSS 28 (SPSS, Chicago, IL, USA) was applied for statistical analysis. As test for normal distribution, the Shapiro–Wilk test was applied. The Spearman’s correlation analysis was used to determine a correlation between the parameters of interest, the chi-square test was applied to compare patient baseline characteristics, and the Wilcoxon–Mann–Whitney test was administered to investigate clinical symptomatology and imaging data before and after surgery. In addition, a step-wise multivariate regression model was applied to investigate independent predictors for the rate of development of spinal cord tethering/syringomyelia. Finally, the *t*-test was applied for investigation of parameters with and without prior trauma surgery. For the purpose of statistical analysis, the level of injury was defined in a consecutive manner beginning with 1 (cervical vertebra 1) up to 24 (lumbar vertebra 5). P-values of lower than 0.05 were regarded as statistically significant.

## Results

In this investigation, 67 patients (56 men, 11 women, mean age 45.8 ± 11.8 years, range 18.1–72.2 years) who received untethering with expansion duraplasty with or without syrinx shunting were included. Most patients (71.6%) were classified as complete spinal cord injury (AIS grade A). The indication for performing untethering surgery was either progressive sensory and/or motor loss in 40.3%, spasticity and/or neuropathic pain in 38.8%, or a combination those symptoms in 20.9% of patients. The complication rate from untethering surgery was 6.0% (hemorrhage and/or shunt dysfunction) and the need for revision (repetition of untethering) was 37.3%. In patients with extensive arachnoiditis (> 3 vertebral segments), the revision rate was higher than in patients with focal arachnoiditis (43.2% vs. 16.7%, p < 0.05). However, in patients with spinal untethering and shunting (as compared to patients with spinal untethering alone), there was no significant difference regarding the revision rate (35.3% vs. 39.4%, p = not significant, NS). An overview of relevant patient baseline characteristics is given in Table 1.

**Table 1:** Baseline characteristics.

	Total (n = 67)	Men (n = 56)	Women (n = 11)	p-value
Age (years)	45.8 ± 11.8	46.5 ± 12.6	42.3 ± 5.6	0.087
Trauma surgery (n, %)	40, 59.7%	33, 58.9%	7, 63.6%	0.771
AIS grade A (n, %)	48, 71.6%	43, 76.8%	5, 45.5%	0.280
Level of SCI (vertebral segments)	11.6 ± 5.4	11.4 ± 5.3	12.7 ± 6.2	0.507
Extent of tethering/syrinx (vertebral segments)	8.1 ± 5.6	8.3 ± 5.6	7.1 ± 5.6	0.560
Interval trauma to untethering (years)	16.8 ± 13.4	17.6 ± 14.0	12.9 ± 9.4	0.187
Revision rate (n, %)	25, 37.3%	22, 39.3%	3, 27.3%	0.451
Complication rate (n, %)	4, 6.0%	4, 7.1%	0, 0%	0.361

*AIS* American Spinal Cord Injury Association Impairment Scale, *SCI* spinal cord injury.

**Figure 1:**
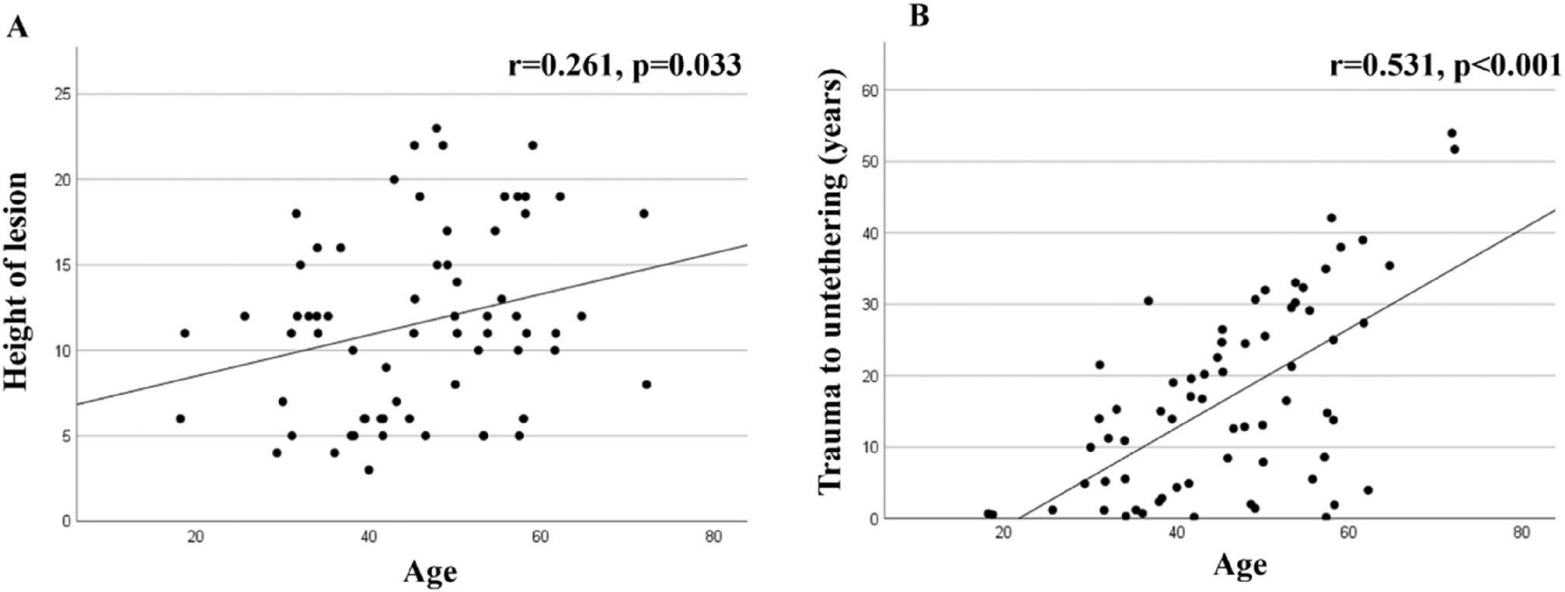
Age-dependent distribution of spinal cord injury (SCI) lesion and interval to untethering surgery (in years). Age significantly correlates with segmental height of the lesion leading to SCI (**A**, p = 0.033) and with the time interval (in years) between initial trauma and untethering surgery (**B**, p < 0.001). Remark on lesion height: vertebral segments were numbered consecutively from the 1st cervical up to the 5th lumbar vertebra (from 1 up to 24).

**Figure 2:**
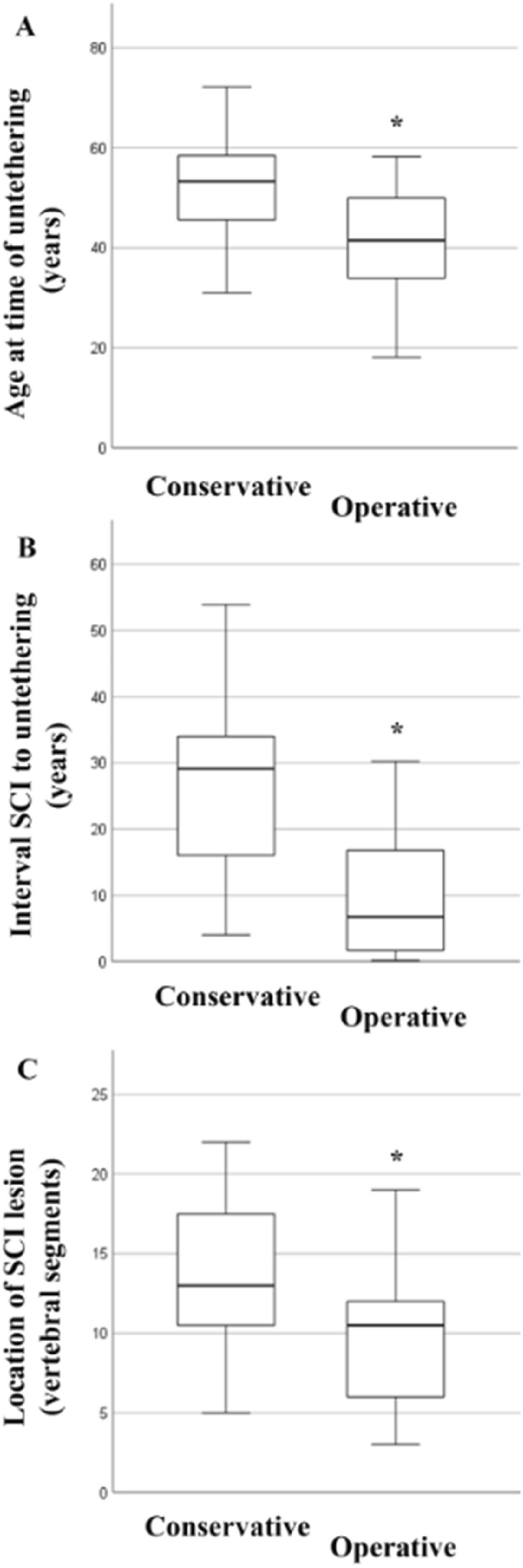
Impact of trauma treatment strategy on spinal cord tethering. (**A**) At the time of untethering surgery, patients who initially received a stabilization of the spine were significantly younger than those patients who were treated conservatively (p < 0.001). (**B**) Further, the interval between spinal cord injury (SCI) up to spinal cord untethering was significantly shorter in patients who received trauma surgery as compared to conservative treatment (p < 0.001). (**C**) Finally, in those patients who underwent initial trauma surgery, the lesion leading to SCI was located significantly higher than in those patients with a conservative treatment (p = 0.016). Remark on lesion height: vertebral segments were numbered consecutively from the 1st cervical up to the 5th lumbar vertebra (from 1 up to 24). Statistically significant differences with a p < 0.05 are marked by an asterisk.

Age is significantly correlated with the level of injury leading to SCI (r = 0.261, p = 0.033, Fig. 1A) and with the time interval between the injury of the spinal cord and untethering surgery (r = 0.531, p < 0.001, Fig. 1B). The extent of tethering and/or syringomyelia was not correlated with age (r = − 0.20, p = NS). Patients with initial trauma surgery (stabilization and/or decompression) were significantly younger (41.7 ± 10.8 years vs. 52.0 ± 10.6 years, p < 0.001, Fig. 2A) while the level of injury was higher (10.5 ± 5.3 vs. 13.3 ± 5.1, p = 0.016, Fig. 2B) than in patients who had received a conservative trauma treatment. Of note, also the time interval between the initial injury of the spinal cord up to the untethering surgery was significantly shorter in patients who received trauma surgery as compared to those patients with conservative treatment (9.7 ± 8.7 years vs. 27.3 ± 12.3 years, p < 0.001, Fig. 2C). Moreover, using the mean age of the patient population as a discriminator, the mean interval between the initial injury of the spinal cord up to the untethering surgery is significantly shorter in younger versus older patients (11.1 ± 9.1 years vs. 22.3 ± 14.7 years, p < 0.001). Therefore, the younger the patient at the time point of trauma and the higher the level of injury, the more rapid the development of symptomatic spinal cord tethering requiring untethering surgery appears to be. The extent of spinal cord tethering and/or syringomyelia was not significantly different between those patients with initial trauma surgery as compared to those with conservative treatment. In a stepwise multivariate regression model adjusted for risk factors potentially promoting arachnoiditis including age, sex, trauma severity (i.e., presence of dural tears, subarachnoid haemorrhage, and/or spinal cord contusion), extent of arachnopathy/syringomyelia, and location of SCI (trauma) lesion, both age (B-coefficient 0.396, p < 0.001) and trauma severity (B-coefficient − 0.462, p < 0.001) were selected as independent predictors for the rate of development of symptomatic tethered cord and syringomyelia while all other variables were excluded from the model.

After performing surgical untethering with expansion duraplasty with or without syrinx shunting, 65.9% of patients showed a significant amelioration of sensory and/or motor disturbances (p < 0.001, Table 2) and 50.0% of patients displayed improvement of spasticity and/or neuropathic pain (p < 0.001, Table 2). In both patient groups with improving symptoms, postsurgical imaging revealed a significant decrease in tethered segments or the extent of syringomyelia (92.1% improvement, p < 0.001). In 26.8% the progression of neurological loss was arrested (p < 0.001, Table 2) and in 45.0% of patients the degree of spasticity and/or neuropathic pain remained on a stable level (p < 0.001, Table 2). Interestingly, postsurgical imaging again displayed a high rate of morphological improvement as compared to the preoperative situation (92.0%, p < 0.001). Finally, 7.3% of patients displayed further progressive neurological loss whereas 5.0% of patients showed worsening of spasticity and/or neuropathic pain (Table 2). Although not statistically significant, postsurgical imaging displayed a 50% amelioration of spinal cord tethering and/or syringomyelia in patient with worsening symptoms after the operation. The AIS grade before and after surgery was not significantly different.

**Table 2:** Surgical outcome in spinal cord tethering and syringomyelia.

	Improved	Stable	Worsened
Sensory and/or motor loss (n, %)	27, 65.9%*	11, 26.8%*	3, 7.3%
Spasticity and neuropathic pain	20, 50.0%*	18, 45.0%*	2, 5.0%
Postsurgical imaging	60, 89.5%*	6, 9.0%	1, 1.5%

Statistical significance (p < 0.001) is marked by an asterisk.

## Discussion

Spinal cord untethering including expansion duraplasty with or without shunting is a successful treatment strategy in patients with symptomatic spinal cord tethering and syringomyelia. About 65.9% of patients demonstrated significant improvement of neurological loss and about 50.0% of patients showed an improvement in spasticity and neuropathic pain. As such, the results of our study corroborate with the existing literature and add further evidence favoring surgical strategies in patients suffering from symptomatic spinal cord tethering and syringomyelia after trauma. In addition, our data reveals that younger age and severe spinal trauma are independent risk factors for development of symptomatic cases. As such detethering strategies in the younger patient risk groups should not be overlooked and if managed early during injury leads to favorable patient outcomes.

**Figure 3:**
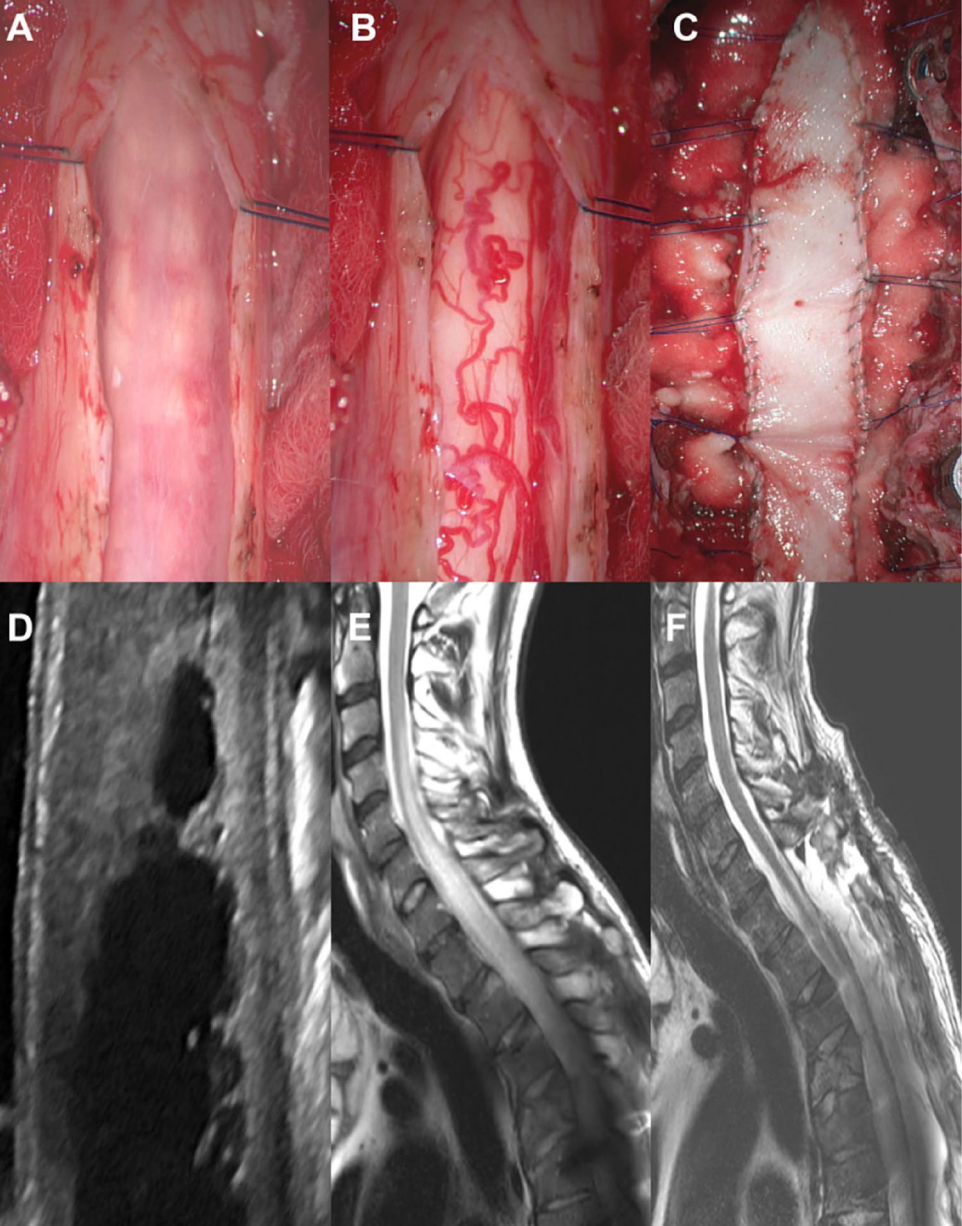
Posttraumatic spinal cord tethering and syringomyelia. (**A**) Midline durotomy is performed with exposure of spinal cord tethering (hypertrophic arachnoid web with scarring aspect and adhesions to the dural sack). (**B**) Posterolateral resection of scarred arachnoid web is conducted under microscopic conditions (lysis of the arachnoid tissue). (**C**) After the completion of the lysis of the posterolateral aspect of the spinal cord, an expansion duraplasty is integrated by continuous and non-resorbable suture and attached to the surrounding tissue or instrumentation material in order to persistently expand the intradural space. (**D**) Intraoperative ultrasound demonstrating the syringomyelia (and spinal cord tethering) is used for intraoperative guidance and quality control. Pre and postoperative magnetic resonance imaging (MRI) show spinal cord tethering and syringomyelia before surgery (**E**) and after untethering and expansion duraplasty (**F**). Note the subtotal collapse of the syringomyelia with persistent augmentation of the intradural space due to expansion duraplasty in panel (**F**).

The optimal therapeutic strategy in patients with spinal cord tethering with or without syringomyelia remains currently a challenge^10^. However, since several years a growing body of evidence points towards surgical untethering of the spinal cord in symptomatic cases^14,15^. In this investigation all patients received spinal cord untethering and expansion duraplasty with or without syrinx shunting (Fig. 3) as described by Falci et al.^5^. The postoperative result of surgical success of untethering and expansion duraplasty was subsequently documented by regular follow up MRI of the spinal axis. Interestingly and in line with the study of Gauillamet et al.^16^, our revision rate in patients with extensive arachnoiditis (> 3 vertebral segments) was significantly higher than in patients with focal arachnoiditis while the need for implantation of a shunt did not affect the rate of surgical revisions. Due to the results of this investigation we are able to further strengthen the level of evidence for recommendation of untethering and expansion duraplasty including cyst shunting (when indicated) with a mean follow-up of 8.1 years after surgery. As such we are in line with one of the largest outcome studies regarding posttraumatic spinal cord tethering and syringomyelia with over 10 years of follow-up using a similar and consistent surgical approach^5^ including an investigation demonstrating good long-term results of a surgical strategy with progressive neurological loss in AIS grade A, B, and E injuries^9^. Of note, postsurgical imaging significantly correlated with improvement of clinical symptoms by a decrease of tethering and collapse of the respective syrinx. Interestingly and on the other hand, a decrease of spinal cord tethering and the collapse of the syrinx in imaging goes not necessarily along with an improvement of but also with an arrest of further deterioration of clinical symptoms. As such, besides the impairment of CSF flow and subsequently conductivity of the spinal cord, the SCI itself plays a critical role in sustaining clinical symptomatology, particularly in a chronic setting of persistent myelopathy. This adheres with a study from Edgar and colleagues who claimed an 87% improvement if untethering surgery is performed within 3 months of onset of clinical symptoms^17^. Thus, a timely surgical intervention in symptomatic spinal cord tethering and syringomyelia helps to omit persistent damage to the spinal cord due to impairment of CSF flow and as such progressive myelopathy. Of note, a promising alternative to untethering and expansion duraplasty has recently been proposed by spine column shortening^18^. Here, the abnormal stretch on the spinal cord due to cord tethering is indirectly treated by a shortening osteotomy, in particular in recurrent tethering or complex cases^19^, and as such might carry less procedural risk as in untethering. However, operation time and blood loss are significantly higher in column shortening osteotomies so that the indication for such an invasive procedure has to be placed carefully.

According to epidemiological studies in SCI cohorts, level and severity of injury significantly correlates with age and male predominance^12^. As such younger patients suffer more frequently from cervical injuries which are associated with higher energy/velocity trauma cases. This is reflected by similar findings in the present patient cohort. Interestingly, age also significantly impacts development of symptomatic spinal cord tethering and syringomyelia in need of surgery. Thus, the younger patients are at the time of SCI, the more rapid clinically relevant spinal cord tethering and syringomyelia develops and consequently and indirectly is also associated with severity of trauma leading to SCI. Conclusively, the extent of mechanical damage to the spinal cord is directly correlated with the velocity of evolution of symptomatic spinal cord tethering. This adheres with recent investigations focusing on trauma severity and inflammatory state^20,21^, which may result in excessive scarring of the subarachnoid web and accelerated development of symptomatic spinal cord tethering and syringomyelia. Notably, several clinical trials targeting mediators of inflammation aim to reduce or omit secondary damage to the spinal cord following SCI and are currently a high intensity research area since the primary damage to the spinal cord can currently not be reversed or repaired^22,23^. Here, B cell lymphocytes might represent a new and promising target in neuromodulation of the central nervous system^24^.

Despite various investigations with large patient numbers favoring surgical untethering of the spinal cord in symptomatic patients, conservative management of posttraumatic spinal cord tethering and/or syringomyelia has been claimed to be most probably equally successful^10^. However and on the one hand, surgical approaches were not concise in those studies and on the other hand, the success of conservative treatment strategies for both progressive neurological loss and/or spasticity/neuropathic pain in SCI patients are frequently double-edged with a host of adverse effects which have been reported to negatively impact quality of life^25,26^. Conclusively, it is important to point out that a standardized surgical approach in symptomatic spinal cord tethering and syringomyelia as based on our results might still provide a valid treatment strategy in this disabling disease without any other efficient treatment alternatives.

As with any other studies, limitations need to be pointed out: first, this is a retrospective investigation at a single treatment center focusing at a rather rare disease which goes along with any sort of bias which can be traced back to such kind of studies. Second, we are not able to provide any sort of quantitative or standardized measurements or scores of pre and postoperative symptomatology, function or outcome as the documentation of the included patients cases was not standardized and due to the changing personnel and documentation systems, data reconstruction was only partially feasible. However, our results are in line with other investigations with higher patient caseloads and as such do not contradict already established findings. Moreover, we consider it highly important to share our experience with this rare pathology, particularly also because of varying results concerning the optimal treatment strategy. Although, over 50% of radiological findings in asymptomatic patients with traumatic SCI are suspicious for the beginning of spinal cord tethering and/or syringomyelia^27–29^, patients without specific symptoms are not routinely referred for further investigation and as such time intervals from SCI to development of spinal cord tethering and from spinal cord tethering to a symptomatic case in need for untethering surgery cannot be reliably provided. Last but not least, it is important to point out that a higher rate of severe trauma in younger patients (with presence of dural tears, subarachnoid hemorrhage, and/or spinal cord contusion) might still be perceived as a potential confounder with regard to the observed correlation of age with the rate of development of symptomatic spinal cord tethering/syringomyelia in need for surgical therapy. Finally, the products used for expansion duraplasty are different across the study cohort. As such, it is not clear whether a part of the revision cases might be explained due to lower biocompatibility of dural expansion products.

## Conclusions

In symptomatic spinal cord tethering and syringomyelia after trauma, surgical untethering with expansion duraplasty provides a promising treatment strategy to recover clinical deterioration in SCI patients. In particular, younger patients with a higher level of SCI are susceptible for a more rapid development of clinically relevant spinal cord tethering and syringomyelia as compared to older patients. Thus, younger SCI patients should undergo a more careful screening with early imaging in order to detect this disabling disease at a most early stage and thus provide optimal and personalized patient care with a reasonable patient outcome.

## Data Availability

The data that support the findings of this study are available from the Swiss Paraplegic Center, Spine and Orthopedic Surgery, but restrictions apply to the availability of these data, which were used for the current study, and so are not publicly available. Data are however available from the authors upon reasonable request and with permission of the local ethics committee (Ethikkommission Nordwest- und Zentralschweiz, EKNZ). The corresponding author can be contacted for guidance concerning the data request.

## References

[1] Krebs J, Koch HG, Hartmann K, Frotzler A (2016). The characteristics of posttraumatic syringomyelia. Spinal Cord.

[2] Yamada S, Zinke DE, Sanders D (1981). Pathophysiology of "tethered cord syndrome". J. Neurosurg..

[3] Fairholm DJ, Turnbull IM (1971). Microangiographic study of experimental spinal cord injuries. J. Neurosurg..

[4] Falci SP (1999). Surgical treatment of posttraumatic cystic and tethered spinal cords. J. Spinal Cord Med..

[5] Falci SP, Indeck C, Lammertse DP (2009). Posttraumatic spinal cord tethering and syringomyelia: Surgical treatment and long-term outcome. J. Neurosurg. Spine.

[6] Lee TT (1997). Progressive posttraumatic myelomalacic myelopathy: Treatment with untethering and expansive duraplasty. J. Neurosurg..

[7] Lee TT, Alameda GJ, Camilo E, Green BA (2001). Surgical treatment of post-traumatic myelopathy associated with syringomyelia. Spine (Phila Pa 1976).

[8] Bonfield CM, Levi AD, Arnold PM, Okonkwo DO (2010). Surgical management of post-traumatic syringomyelia. Spine (Phila Pa 1976).

[9] Klekamp J (2012). Treatment of posttraumatic syringomyelia. J. Neurosurg. Spine.

[10] Kleindienst A, Laut FM, Roeckelein V, Buchfelder M, Dodoo-Schittko F (2020). Treatment of posttraumatic syringomyelia: Evidence from a systematic review. Acta Neurochir. (Wien).

[11] Li YD, Therasse C, Kesavabhotla K, Lamano JB, Ganju A (2021). Radiographic assessment of surgical treatment of post-traumatic syringomyelia. J. Spinal Cord Med..

[12] Toda M, Nakatani E, Omae K, Fukushima M, Chin T (2018). Age-specific characterization of spinal cord injuries over a 19-year period at a Japanese rehabilitation center. PLoS ONE.

[13] Devivo MJ (2012). Epidemiology of traumatic spinal cord injury: Trends and future implications. Spinal Cord.

[14] Stenimahitis V, Fletcher-Sandersjoo A, Tatter C, Elmi-Terander A, Edstrom E (2022). Long-term outcome following surgical treatment of posttraumatic tethered cord syndrome: A retrospective population-based cohort study. Spinal Cord.

[15] Holmstrom U, Tsitsopoulos PP, Flygt H, Holtz A, Marklund N (2018). Neurosurgical untethering with or without syrinx drainage results in high patient satisfaction and favorable clinical outcome in post-traumatic myelopathy patients. Spinal Cord.

[16] Guillaumet G (2021). Reintervention rate of arachnolysis versus shunting for nonforaminal syringomyelia. J. Neurosurg. Spine.

[17] Edgar R, Quail P (1994). Progressive post-traumatic cystic and non-cystic myelopathy. Br. J. Neurosurg..

[18] Lin W (2018). Spine-shortening osteotomy for patients with tethered cord syndrome: A systematic review and meta-analysis. Neurol. Res..

[19] McVeigh LG, Anokwute MC, Chen S, Jea A (2022). Spinal column shortening for tethered cord syndrome: A systematic review and individual patient data meta-analysis. J. Neurosurg. Pediatr..

[20] Kwiecien JM (2021). The role of astrogliosis in formation of the syrinx in spinal cord injury. Curr. Neuropharmacol..

[21] Kwiecien JM (2020). Prolonged inflammation leads to ongoing damage after spinal cord injury. PLoS One.

[22] Khaing ZZ (2023). Clinical trials targeting secondary damage after traumatic spinal cord injury. Int. J. Mol. Sci..

[23] Wang YT, Lu XM, Chen KT, Shu YH, Qiu CH (2015). Immunotherapy strategies for spinal cord injury. Curr. Pharm. Biotechnol..

[24] Maheshwari S, Dwyer LJ, Sirbulescu RF (2023). Inflammation and immunomodulation in central nervous system injury—B cells as a novel therapeutic opportunity. Neurobiol. Dis..

[25] Dietz N, Wagers S, Harkema SJ, D'Amico JM (2023). Intrathecal and oral baclofen use in adults with spinal cord injury: A systematic review of efficacy in spasticity reduction, functional changes, dosing, and adverse events. Arch. Phys. Med. Rehabil..

[26] Mei L (2022). Efficacy and safety of different drug treatments in patients with spinal-cord injury-related neuropathic pain: A network meta-analysis. Spinal Cord.

[27] Backe HA, Betz RR, Mesgarzadeh M, Beck T, Clancy M (1991). Post-traumatic spinal cord cysts evaluated by magnetic resonance imaging. Paraplegia.

[28] Silberstein M, Hennessy O (1992). Cystic cord lesions and neurological deterioration in spinal cord injury: Operative considerations based on magnetic resonance imaging. Paraplegia.

[29] Milhorat TH, Johnson RW, Milhorat RH, Capocelli AL, Pevsner PH (1995). Clinicopathological correlations in syringomyelia using axial magnetic resonance imaging. Neurosurgery.

